# The role of estimated muscle power from a sit-to-stand test in determining frailty in community-dwelling older adults

**DOI:** 10.1371/journal.pone.0352160

**Published:** 2026-07-02

**Authors:** Cillin Condon, Eleanor Fallon, Fearghal P. Behan, Roman Romero-Ortuno, Seán McAuliffe

**Affiliations:** 1 Discipline of Physiotherapy, School of Medicine, Trinity College, Dublin, Ireland; 2 School of Allied Health, University of Limerick, Limerick, Ireland; 3 Discipline of Gerontology, School of Medicine, Trinity College, Dublin, Ireland; 4 Ageing Research Centre, Health Research Institute, University of Limerick, Limerick, Ireland; University of Naples Federico II, ITALY

## Abstract

**Introduction:**

This study explored the usefulness of estimated lower-limb muscle power, derived from the 5-times sit-to-stand (5xSTS) test, for identifying frailty among community-dwelling older adults in Ireland.

**Methods:**

Data from Wave 3 of The Irish Longitudinal Study on Ageing (TILDA) were analysed, focusing on adults aged 50 years and older. Muscle power was estimated using a standardised equation from the five-time sit-to-stand (5xSTS) test, incorporating body height, mass, and chair height. Frailty status was classified using an Index, according to established criteria. Logistic regression models assessed the predictive capacity of muscle power relative to 5xSTS. Thresholds for frailty risk were explored through Receiver Operating Curves and Locally Weighted Scatterplot Smoothing.

**Results:**

Findings reveal a decline in muscle power with advancing age, more pronounced in females and frail individuals. Muscle power estimates showed moderate agreement with frailty status, with sensitivity and specificity comparable to those of the 5xSTS. Muscle power less than 2.5 Watt·kg ^-^¹ in males and 2.08 Watt·kg ^-^¹ in females was associated with increased frailty risk, consistent with other studies. Overall power estimation showed a predictive performance similar to that of traditional assessments such as Timed Up and Go, supporting its utility in clinical and community settings.

**Conclusion:**

Estimated muscle power derived from the 5xSTS test is a practical, reliable tool for early identification of frailty among older adults. Its accessibility and predictive validity suggest it could complement existing clinical assessments but not replace them.

## Introduction

The proportion of older adults worldwide is expected to double, from 12% to 24%, between 2015 and 2050 [1]. Population ageing can be seen as one of the greatest successes of public health. However, increased life expectancy presents challenges in ensuring those extra years are healthy and disability-free.

With chronological age, there is a decline in skeletal muscle strength (the amount of force a muscle can produce with a single maximal effort) and power (the ability to exert maximal force in a short time), with power declining more rapidly than muscle strength [2–5]. Skeletal muscle mass and strength decline gradually by 2% each year from the age of 60 [6] with more pronounced age-related loss of power, declining by 3–3.5% annually after the 6^th^ decade [7, 8]. Studies have demonstrated that lower power output during functional tests, such as sit-to-stand (STS), is significantly associated with higher frailty levels, greater fall risk, and adverse conditions [9,10]. Although related, muscle strength and power are distinct parameters, and studies have found that muscle power may be a more discriminatory predictor of overall functional performance in older adults [11, 12]. Not only is power functionally important, but it is also a key determinant of adverse outcomes such as morbidity, disability, and frailty [13, 9, 8].

Frailty has received much attention as an extreme consequence of ageing and is associated with loss of muscle strength and power. Frailty is a clinical syndrome marked by decreased physiological reserve and increased vulnerability to adverse health outcomes such as falls, disability, hospitalisation and mortality [10]. The deterioration of physical function in older adults is a precursor stage of frailty [14,15] and a major domain examined in most frailty assessment tools [2]. Various Frailty Scales have assessed different aspects of physical performance, such as muscle strength and gait speed [6]. However, a growing body of evidence suggests that the decline in power occurs earlier than strength, and this may have a greater impact on function than previously considered [3]. Different researchers have suggested that power may be a better predictor of adverse events than traditional measures such as grip strength or timed gait tasks [15]. The role of power (a physical domain) in identifying a multidimensional construct such as frailty has only been recently explored, i.e. using an Index to identify sarcopenia [16].

In the absence of cheap objective tools, estimates of lower limb power may be utilised [12]. Researchers have identified a power threshold below which individuals are more likely to be classified as frail or pre-frail [7]. Because the ability to generate sufficient power is essential for quick and safe transitions from sitting to standing or reacting to perturbations—actions critical to daily living—a low power output indicates compromised functional reserve [17–19]. Recent evidence suggests that estimating power from repeated sit-to-stands actions, either as time to complete five full stands (5xSTS) or the number of repetitions completed in 30 secs (30STS), is feasible in clinical settings using simple equipment and standardised equations [7, 11, 20,21,22,23]. However, other studies have not found this estimation superior at identifying fallers or fractures among older persons [24]. STS-estimated power may enhance the multidimensional assessment of frailty compared with current methods such as gait speed, TUG, and handgrip, which are currently recommended for sarcopenia evaluation [25].

The primary objective of this study is to estimate power in males and females aged 50 + in an Irish population and to compare these results to international cohorts

The secondary objective is to examine the role of power in identifying frailty compared with the 5 times Sit-to-Stand test.

## Methods

The study population was from Wave 3 of The Irish Longitudinal Study on Ageing (TILDA), a nationally representative cohort study on community living adults over 50 years and older in the Republic of Ireland [26] between March 2014 and October 2015. This study included: (i) a computer-assisted personal interview (CAPI); (ii) a self-completion questionnaire (SCQ); and (iii) a comprehensive health assessment [27]. Further information can be found in previously published literature [28]. The Tilda study was accessed on 6/6/2025, and no information that could identify individual participants was available during or after data collection

### Assessment of frailty

Frailty was determined using a Frailty index (FI) based on a deficit accumulation model, (e.g., symptoms, diseases, impairments, or test results) [29]. The index is created by summing deficits (0, 0.5, or 1) divided by the number of items, yielding a score range from 0 to 1. Frailty status was classified as non-frail (0–0.1), pre-frail (0.11–0.24) and frail (≥0.25). This process has been used in previous studies [30–32]. The FI utilised 22-items from the TILDA dataset (S6 File). These include 20 self-reported items and an additional two physical parameters used in other Frailty scales (grip, TUG speed).

### Health assessment

Participants in the TILDA study had either a health assessment at home or at a designated health centre [26]. In addition to the CAPI and SCQs, the following performance tests were conducted:

- **Height:** measured with a Seca 240 wall-mounted stadiometer (without shoes or heavy clothing).
- **Mass:** measured to the nearest 0.1 kg using a SECA electronic floor scale (without shoes or outerwear).
- **Body Mass Index (BMI):** calculated as mass (kg) divided by height (m²).
- **Grip Strength:** assessed with a handheld dynamometer using a standardised protocol.
- **Timed Up and Go (TUG):** participants stood from a chair, walked three metres, turned, returned, and sat down; time to complete was recorded in seconds.
- **Five Times Sit-to-Stand (5xSTS):** used as an indicator of lower-limb strength and power. Participants were first tested for their ability to stand once without arm support. Those who succeeded were timed while performing five consecutive sit-to-stand movements with arms crossed over the chest. Only one trial was performed. Chair height was standardised in the health centre and measured for home visits [26].

### Estimated muscle power

Estimated muscle power was calculated using the equation from Alcazar et al [22].


$$\begin{array}{c} \text{Relative STS Power }(\text{Watts})\;=\;\frac{\text{Body Mass }(\text{kg})\;\times \;\text{0}.\text{9}\;\times \text{ g }\times \;\left[\text{Height }(\text{m})\;\times \;\text{0}.\text{5}\;-\text{ Chair height }(\text{m})\right]}{\text{Five STS time }\times \;\text{0}.\text{1}} \end{array}$$

Then normalised to body mass:


$$\begin{array}{c} Watt.{Kg}^{-\text{1}}\;=\;Relative\;STS\;Power/\;Body\;Mass \end{array}$$

### Statistical analysis

All data were analysed in SPSS 29. Data was examined for normality with the Shapiro-Wilk test. Standard descriptive statistics were used for continuous variables and frequency tables for categorical variables. Mann-Whitney tests were used to compare groups where appropriate. Chi-square tests were used to compare Frailty status by gender. Binary logistic regression was used to assess the impact of power on the ability to identify frail or non-frail individuals according to the FI. The prefrail group was not included at this time, as the discriminatory ability of the estimated muscle power has not been established; including the prefrail group would be premature until this has been assessed.

A second model was also run with 5xSTS time to determine the impact of muscle strength on the identification of frail or non-frail individuals (excluding pre-frail). A likelihood-ratio test compared the power model to the 5xSTS Time model. The power model contained variables: Age, Sex, BMI, Dominant Grip Strength, and estimated muscle power (Watt.Kg^-*1*^), while the 5xSTS model replaced power with Sit-to-Stand time (secs). Previous research has demonstrated positive associations between lower STS times and lower limb strength or power [2,7,33]. The variables of interest (5xSTS time and Power) were not included in the same model to mitigate multicollinearity (measured by Variance Inflation Factor).

Receiver operating characteristic (ROC) curves were also used to assess the diagnostic value of estimated muscle power in identifying frail individuals. For each cutoff, sensitivity (true-positive rate), specificity (true-negative rate), positive predictive value (PPV), negative predictive value (NPV), and overall classification accuracy were computed. Finally, we used locally weighted scatterplot smoothing (LOWESS), ROC analysis, and the Area under the Curve (AUC) to explore the relationship between predicted frailty status and muscle power (Watt.Kg^-1^) and to identify potential cut-off points for changes in frailty risk.

### Ethical statement

Formal ethics for this study was not required as the data is publicly available and participants provided written informed consent on enrollment to the study. TILDA data are fully anonymised and publicly accessible through the Irish Social Science Data Archive (ISSDA) at University College Dublin (http://www.ucd.ie/issda/data/tilda/). Data were used solely for this research purpose in compliance with the access agreement and ethical standards governing its use.

## Results

### Participant characteristics

TILDA is a prospective cohort study of 8,175 community-dwelling adults aged 50 years and older. This study was based on a sample of participants for whom results from the 5xSTS outcomes and other physical tests were available. This sample was 4,295 (1,905 males (44%), 2,390 females (56%). The largest proportion of the study sample involved individuals aged in their 60s (40.4%, n = 1,737) followed by participants in their 50s (30.2%, n = 1,297), 70s (22.3%, n = 957), and the 80s and older (7.1%, n = 304). This distribution reflects a predominantly younger-older adult population, with over 70% of the sample under 70. Using a Frailty Index with previously published cut-off points [30], 2,172 participants were defined as non-frail (50.6%), 1,452 (33.8%) pre-frail, and 671 (15.6%) frail, respectively.

Females were significantly more likely to be classified as frail and pre-frail and demonstrated lower functional performance across all measures compared to males. Specifically, female participants had lower grip strength, slower chair-stand times, and reduced power across all age groups (Table 1, p < 0.001). Power declined with age in both male and female cohorts, but it was consistently higher in males (Table 1). Although differences in mean Timed Up and Go (TUG) times were statistically significant (p < 0.001), the times were comparable between sexes. Overall, a significantly greater proportion of men were categorised as non-frail, whereas frailty was more prevalent among women (p < 0.001) (Table 1). Spearman’s rank-order correlations (S5 Table) indicated that Frailty Index (FI) had only a moderate positive association with TUG time (ρ = 0.45, p < .001) and low to fair correlation with 5XSTS time (ρ = 0.31, p < .001), and had a weak negative association with Grip Strength (ρ = –0.36, p < .001) and Muscle Power (ρ = –0.37, p < .001).

**Table 1:** Participant demographics.

	Male	Female	Total
*n =*	1,905 (44.4%)	2,390 (55.6%)	4,295 (100%)
*50s*	530 (27.8%	767 (32.1%)	1,297 (30.2%)
*60s*	772 (40.5%)	965 (40.4%)	1,737 (40.4%)
*70s*	468 (24.6%)	489 (20.5%)	957 (23.3%)
*80 + s*	135 (7.1%)	169 (7.1%)	304 (7.1%)
*BMI Median (IQR)*	28.5 (5)	27.2 (7)	
*Muscle Power 50s Mean (SD)*	3.0 (0.65)	2.5 (0.60) *	
*Muscle Power 60s Mean (SD)*	2.9 (0.69)	2.3 (0.69) *	
*Muscle Power 70s Mean (SD)*	2.6 (0.61)	2.1 (0.55) *	
*Muscle Power 80s Mean (SD)*	2.4 (0.65)	1.9 (0.41) *	
*5xSTS Time (sec) Median (IQR)*	12.9 (4)	13.1 (4) *	
*Grip (Kg) Median (IQR)*	35.0 (11)	21.5 (7) *	
*TUG (sec) Median (IQR)*	8.8 (2)	8.7 (2) *	–
*Frailty Index Mean (SD)*	0.12 (0.09)	0.14 (0.11) *	–
*Non frail (%)*	1,031 (54.1%)	1,141 (46.7%)	2,172 (50.6%)
*Pre frail (%)*	640 (33.6%)	812 (34.0%) *	1,452 (33.8%)
*Frail (%)*	234 (12.3%)	437 (18.3%) *	671 (15.6%)

Data presented as n (%), mean SD or median (IQR).

*Significant difference between males and females (Mann-Whitney test/*X*^2^), p < 0.05.

**Fig 1:**
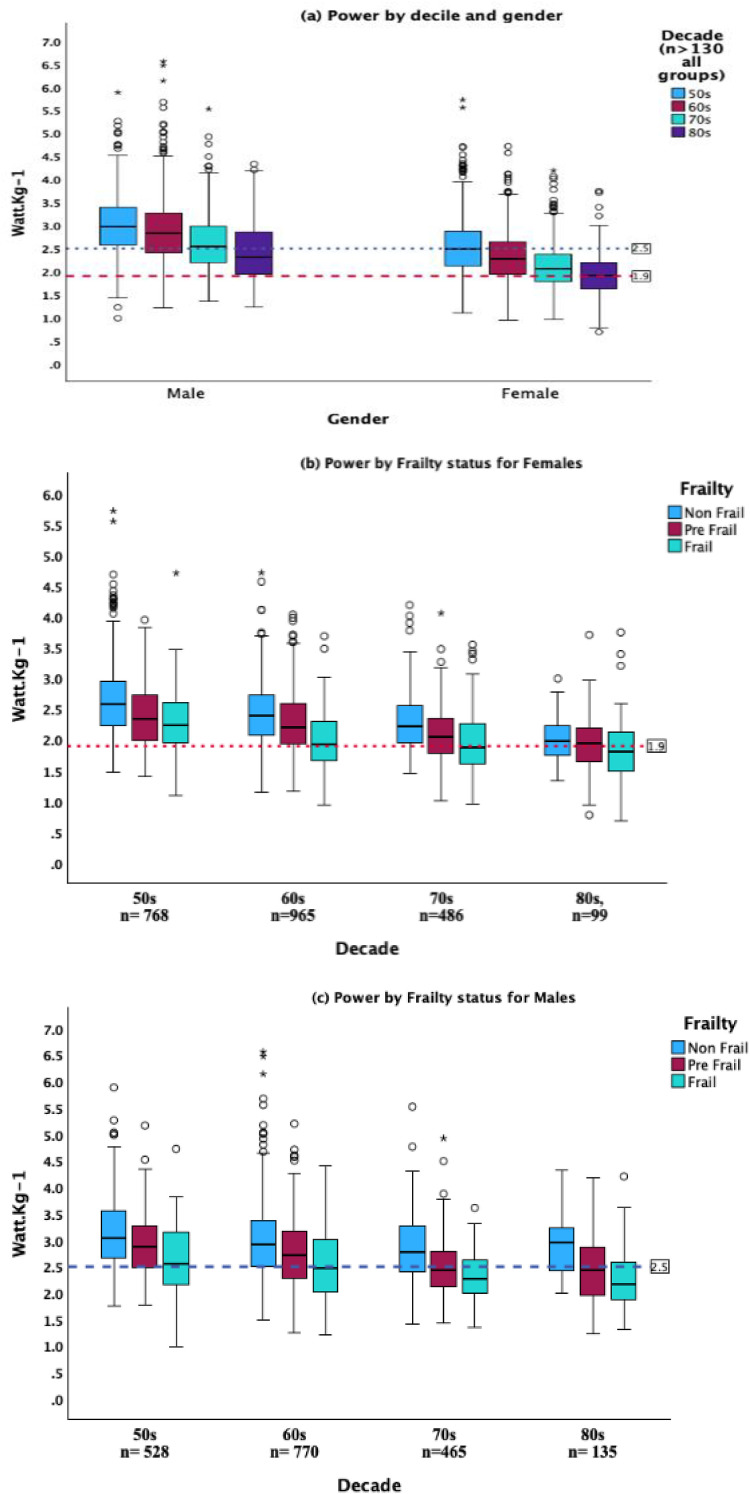
Muscle power by decile and frailty status.

Fig 1(a) illustrates the change in power in males and females across the decades, with the decline more pronounced after the 7^th^ decade. Fig 1(b), (c) show the power for females and males by frailty state within each decile. The decline in mean power from the 50s to the 80s was −0.62 Watt.Kg^-1^ for the non-frail, −0.46 Watt.Kg^-1.^ for the pre-frail and −0.41 Watt.Kg^-1^ for frail females. For males, the decline in mean power in the same categories was – 0.21 Watt.Kg^-1^ (non-frail), −0.44 Watt.Kg^-1^ (pre-frail) and −0.36 Watt.Kg^-1^ (frail). The caveat is that this is a cross-sectional study, and there are relatively low numbers of frail persons in their 50s. The horizontal lines red (female) and blue (male) in Fig 1 include the cut-off points for frailty proposed by Alcazar et al.[7,23]. Supplemental data include the centiles for each decile, gender, and frailty state (S2 and S3 Tables).

### Logistic regression models for frailty prediction

Two logistic regression models (Power vs 5xSTS) were developed to identify a binary state of Frailty (Frail versus Non-Frail) among 2,593 individuals, using two sets of physiological and functional performance variables (Table 2). Both models demonstrated good discriminative performance (AUC > 0.85) [34] and differed only slightly in their predictive value and in the interpretation of frailty status (frail vs non-frail). Both models demonstrated excellent fit with high overall explanatory power. The 5xSTS model slightly outperformed the power model in terms of model likelihood and R^2^, but the differences were negligible (S4 Table). Both models had high specificity but only moderate sensitivity. The power model had a sensitivity of 54.8% for frailty status and a specificity of 95.9% for non-frailty, while the 5xSTS model reported similar sensitivity (55%) and specificity (96.1%). The power model and the 5xSTS model demonstrated comparable classification accuracy for identifying frailty or non-frailty, with no statistically significant difference between them (86.4% vs 86.6%) (Table 2).

**Table 2:** Binary logistic regression predictors of frailty status. Predictor Effects (Odds Ratios).

Predictor	(Muscle Power) Adjusted OR (95% CI)	(STS Time) Adjusted OR (95% CI)
Age	0.97 (0.95-0.98)*	0.97 (0.95-0.98)*
BMI	0.92 (0.89-0.93)*	0.91 (0.88-0.93)*
Grip Strength (Dominant Hand)	1.11 (1.08-1.13)*	1.11 (1.08-1.13)*
Female Gender	4.09 (1.3-13.0)**	3.23 (1.2 −8.8)**
TUG Time	0.58 (0.53-0.63)*	0.56 (0.54-0.65)*
5xSTS	N/A	0.89 (0.83-0.94)*
Power	1.83 (1.31-2.54)	N/A
Gender X Power	0.82 (0.52-1.28)^**$**^	0.83 (0.54-1.24)^**$**^

**Note:** OR = Odds Ratio; TUG = Timed Up and Go Test. Dependent variable: Frailty (1 = Frail, 0 = Non-frail). *Significant at p < .001, ** Significant at p < .05, $ Non-significant.

Both models significantly predicted Frailty with a good fit. Muscle power demonstrated a slightly better explanatory effect (−2 Log Likelihood = 1821.26.96, Cox and Snell R^2^ = .32, Nagelkerke R^2^ = .48, compared with 5xSTS (−2 Log Likelihood = 1816.53, Cox and Snell R^2^ = .32, Nagelkerke R^2^ = .48, Hosmer–Lemeshow χ²(8) = 12.95, p = .114). Predictive accuracy was nearly identical, with muscle power performing marginally better (0.02%).

Across both models, higher TUG time remained one of the strongest predictors of frailty. Better grip strength is consistently associated with a lower risk of frailty. Higher BMI and increasing age are consistently associated with frailty (i.e., the inverse of non-frailty). Being female was strongly associated with frailty (OR 4.5). However, the interaction between gender and muscle power was not significant (OR 0.82, CI 0.52–1.28, p = 0.38), indicating that the protective effect of muscle power did not differ significantly between males and females.

#### Diagnostic accuracy of power for identifying frailty.

**Fig 2:**
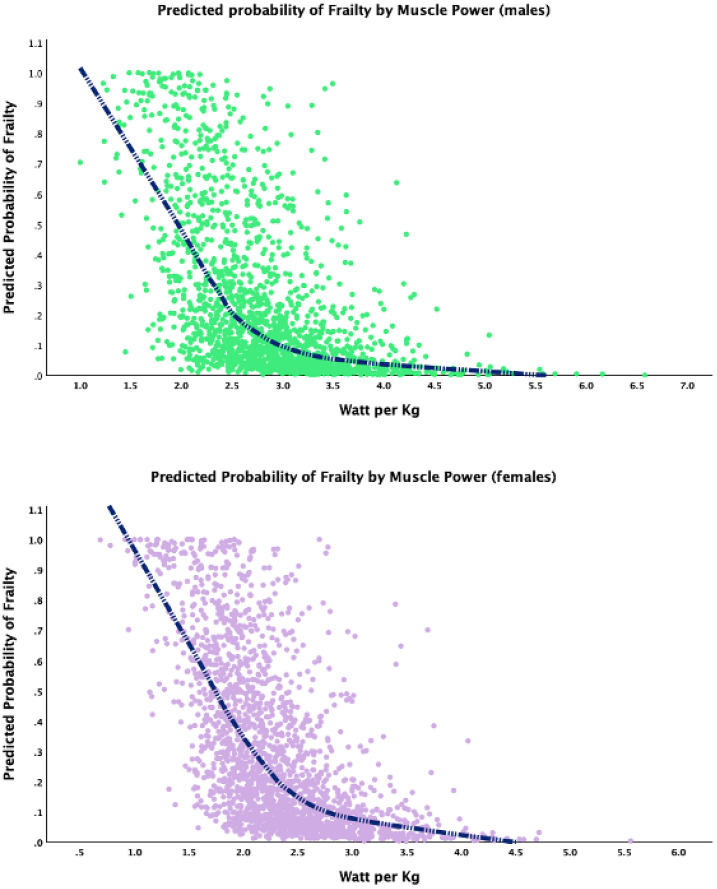
Predicated Frailty by power.

To explore the proposition of cutoffs for defining frailty, described by Alcazar et al.[7], a two-way scatter plot was created to show predicted frailty against the continuous power variable (Fig 2) for both males and females. The figure shows a decline in predicted frailty with increasing muscle power. The optimal cutoff based on the Youden Index, where the chance of being frail drops significantly, for men, was > 2.50 Watt.Kg-^1^, yielding a sensitivity of 77.2% and a specificity of 61.4%. For women, the optimal cutoff was > 2.07 Watt.Kg-^1^, with a sensitivity of 77.6% and a specificity of 60.4%.

A receiver operating characteristic (ROC) analysis was conducted to evaluate the ability of power to identify frailty / non-frailty classification. For men, the ROC curve demonstrated a statistically significant ability to discriminate frail from non-frail participants, with an AUC of 0.75 (95% CI: [0.71–076 CI], p < .001). For females, the AUC was 0.74 (95% CI: [0.71–0.77], p < .001), also indicating moderate discrimination. Overall, these results suggest that power is a useful predictor of frailty in both men and women, with similar diagnostic accuracy across genders.

## Discussion

The primary objective of this study was to characterise muscle power in a large cross-sectional, community-dwelling Irish population. Males had greater power than females across all age groups and frailty states. The findings show a decline in power with age in both males and females, with the decline more pronounced in females. Declines in power were more pronounced after the 7th decade in both males and females, with the decline greater in individuals categorised as frail or prefrail (S2 and S3 Tables). The secondary objective of the current study was to identify a binary frailty state (Frail or Non-Frail) using estimates of lower limb power, rather than traditional methods (5xSTS). Both methods, using logistic regression models, demonstrated excellent fit, high explanatory power, and similar performance, with high specificity but only moderate sensitivity in identifying frailty status in older adults. Because estimated power was shown to be similar to 5xSTS, we explored the impact of specific cutoffs for power to determine frailty status. A plot of predicted frailty against power illustrated a reduced risk of becoming frail at values < 2.5 Watt.Kg-1 for males and < 2.08 Watt.Kg-1 for females, similar to those published elsewhere [7]. This strengthens the case for a minimum threshold for functional capacity in the lower limbs [18,35], but the optimal method for clinically identifying this threshold remains unclear.

In the current TILDA dataset, the median power for non-frail females ranged from 2.0 to 2.4 Watt.Kg^1^, with values of 2.92 to 2.95 Watt.Kg^-1^ reported for males. These findings broadly agree with the literature, which shows that the current cohort has a similar power profile, albeit slightly lower than that of their European peers. Studies by Alcazar et al [7,36] compared power in older adults across four European cohorts. Using estimated power from the 30-second STS test, they reported an overall value of 2.7–3.2 Watt.Kg^-1^ for females and 3.5–4.2 Watt.Kg^-1^ for males [7]. The higher power values observed in this broader European cohort may be attributed to the duration of the 30xSTS test used, whereas the current study used the time to complete 5 sit-to-stands. However, Baltasar-Fernandez et [37] also utilised the 5xSTS test to estimate muscle power in adults >65 years and found power values comparable to our data, with 2.1 and 2.7 Watts.Kg^-1^ for females and males. It is acknowledged that a single physical test is not definitive for assessing frailty, but physical tests can be used alongside other clinical tools [38,39]. Losa Reyna et al [40] showed that low relative sit-to-stand (STS) muscle power (female <1.7 Watt·kg^−^¹, male <2.2 Watt·kg^−^¹) is significantly associated with frailty (defined using Fried's criteria and the Frailty Trait Scale) in older adults and suggested it provided greater clinical relevance than traditional sarcopenia criteria like grip strength. Alvarez Bustos et al [23] used power to identify frailty derived from the 5 times STS test in a range of settings. They used eight different frailty scales because there was no universally recognised gold standard. Power was able to predict frailty (AUCs > 0.7) across all measures with Odds Ratios ranging from 0.14 to 0.46. Our study reported an odds ratio of 1.83 for muscle power predicting frailty, which, when inverted (1/1.83 = 55%), means that for each unit increase in power (1 Watt·kg^−^¹), the chance of being frail is reduced by 55%. Although it should be noted that changes in the order of 0.1–0.2 are more likely to occur.

In our study, 55% of both males and females were between 2 and 3 Watt.Kg^-1^ Similarly, Burbank et al. demonstrated that power had significant predictive value for identifying frailty among older adults in a 4-year prospective study in the USA [11]. Participants with lower power (<median 231.2 Watts) who were pre-frail or non-frail at baseline had a higher risk of becoming frail. In contrast to these findings, Kirk et al [24] showed power (<1.6 Watt.Kg^-1^ for females, < 2.0 Watt.Kg^-1^ for males) had poor ability in identifying persons with a history of falls or fractures, AUC (<0.65). As a tool for non-frailty states, Alcazar et al [7], reported an AUC > 0.85 for estimated power as a measure for identifying mobility-limited older adults, defined by slow gait speed or TUG.

In terms of clinical utility, power (Watt or Watt· kg-^1^) is not commonly used in ageing populations, whereas it is routinely assessed in athletic populations. Reviews of power training in healthy older adults have consistently shown it to be beneficial and safe, but more work needs to be done in populations with chronic disease [3]. One difficulty in determining whether power plays a role in defining or measuring frailty is that frailty incidence depends on the classification tool used [31]. In defining frailty, we recreated, as far as possible, the Frailty Index used by others [30, 23]. As agreement on the assessment of frailty and its precursor (pre-frailty) is developing, the need to develop tools that accurately measure the elements of frailty and their trajectory remains [41]. Whilst this study limited itself to a binary approach (frail vs non-frail), the role of muscle power in the pre-frail group would be of interest to follow over time, to determine whether pre-frail persons develop frailty or can reverse to non-frail status, as this may confer greater health benefits for the ageing population. Longitudinal studies, such as the TILDA study, can assess this over time.

Whilst estimated power has limited utility for defining frailty, it may offer a more robust measure of its reversibility. Romero-Ortuno et al have demonstrated that frailty states are not fixed [42]. Exercise and dietary protein interventions have shown that the risk of frailty can be reduced by half [43]. The stressor or event that tips someone from a state of health into frailty may be reversed, and with this, restoring physiological reserve [44]. Whitson et al. have argued that physiological reserve or resilience is not the opposite of frailty but a different form of the construct [45], and thus may require novel measurement. Although a 1 Watt.Kg^-1^ gain is a relatively large gain; the incremental scale on which power is measured may be a more sensitive measure of physiological resilience or recovery over traditional methods such as TUG or STS time (seconds). Another factor to consider is BMI. The median BMI was 28.1 (males) and 27.5 (females) in this population. The role of sarcopenic obesity, characterised by low muscle mass and excess fat, in the assessment of muscle power has yet to be fully explored [46]. Adjustments to the Watts per body mass, taking into account muscle mass or fat mass, may improve the sensitivity of muscle power. Alcazar et al [7] suggested a Minimal Clinically Important Difference (MCID) of 0.33 Watt.Kg^-1^ in women and 0.42 Watt.Kg^-1^ in men but this needs to be validated in further rehabilitation studies. Kim and Rockwood have proposed a framework to prevent and manage frailty, promoting increased physiologic reserve in prefrail or frail individuals to build robustness and resilience [47]. Muscle function declines with chronological age for many reasons, and many approaches have been proposed to reverse or arrest this decline and prevent the ‘natural’ decline from becoming a functional loss [48]. Efforts to identify those crossing a ‘tipping point’ for conditions such as sarcopenia or frailty should be explored [49]. Power training can be considered an additional tool for increasing physiological reserve [50]. Twelve weeks of exercise can improve power by 0.53–0.82 Watt.Kg^-1^ in older adults [51]. As the accuracy of estimated power is further tested for reliability, or direct power measures are improved with technology [52], the current timed test measures (TUG or STS) remain the clinical standard until objective, easy-to-use tools for power measurement are developed for routine clinical practice [53].

## Limitations

A direct comparison with other published frailty measures was not possible, as the TILDA data lacked objective measures of balance. Gait speed was calculated from the TUG, which has a shorter distance than other gait measures, e.g., the 4 m walk. Furthermore, alternative approaches to define cut-offs for the FI were available and may have been more appropriate for a ‘young older adult’ sample [54]. It is acknowledged that tests such as the sit-to-stand or TUG use only a small percentage of a person's capacity. Thus, any extrapolation of lower limb power is based on submaximal tests. This can be beneficial for individuals who may not be able to complete more demanding tests, but it does create a ceiling effect for more physically able people. The power presented here is still an estimate, based on individuals’ physical performance, using indirect equations rather than a direct measure of the muscles involved in the sit-to-stand action. These are closely related (supplemental data, r = −0.86, increased chair stand times are highly but negatively correlated with estimated power) and not fully independent constructs.

Future work measuring power using dynamometry or force plates may add greater precision to current measures in the literature, but it introduces logistical barriers in large-sample longitudinal cohorts and clinical settings. Furthermore, the cut-off points used for comparison have been published for populations [7,40] described as either limited in mobility or frail, using different scales; direct comparisons are not possible but are inferred.

## Conclusion

In summary, estimated power did not significantly outperform STS chair stand time in identifying frailty within an Irish population. Power declined across the decades, in line with European studies, and the cut-off points of <2.1 and <2.5 W.Kg^-1^ for females and males, respectively, were found to be valid in an Irish population as part of the efforts to identify frailty.

## Supporting information

S1 Table Frailty by decile & gender. (DOCX)

S2 Table Power decade and frailty status (Women). (DOCX)

S3 Table Power by decade and frailty status (Men). (DOCX)

S4 Table Performance of models. (DOCX)

S5 Table Correlations between frailty and physical measures. (DOCX)

S6 File Frailty index. (DOCX)

S7 File Sensitivity analysis. (DOCX)
