# Supplementary material for: The role of estimated muscle power from a sit-to-stand test in determining frailty in community-dwelling older adults
Source: PLoS One. 2026 Jul 2;21(7):e0352160. doi: 10.1371/journal.pone.0352160 (PMC13327205; doi:10.1371/journal.pone.0352160)
Supplement: S1 Table — (DOCX) [file pone.0352160.s001.docx]

**S1 Table 1 . Frailty by decile & gender**

|  | Male   non-frail | Male   Pre Frail | Male Frail | Female   non-frail | Female   Pre Frail | Female Frail | Total n= (%) |
| --- | --- | --- | --- | --- | --- | --- | --- |
| 50s | 364 | 126 | 40 | 476 | 205 | 86 | 1297 |
|  | 8.5% | 2.9% | 0.9% | 11.1% | 4.8% | 2.0% | 30.2% |
| 60s | 460 | 239 | 73 | 493 | 321 | 151 | 1737 |
|  | 10.7% | 5.6% | 1.7% | 11.5% | 7.5% | 3.5% | 40.4% |
| 70s | 193 | 204 | 71 | 144 | 213 | 132 | 957 |
|  | 4.5% | 4.7% | 1.7% | 3.4% | 5.0% | 3.1% | 22.3% |
| 80s | 14 | 71 | 50 | 28 | 73 | 68 | 304 |
|  | 0.3% | 1.7% | 1.2% | 0.7% | 1.7% | 1.6% | 7.1% |
|  | 1031 | 640 | 234 | 1141 | 812 | 437 | 4295 |
