# Supplementary material for: The role of estimated muscle power from a sit-to-stand test in determining frailty in community-dwelling older adults
Source: PLoS One. 2026 Jul 2;21(7):e0352160. doi: 10.1371/journal.pone.0352160 (PMC13327205; doi:10.1371/journal.pone.0352160)
Supplement: S2 Table — (DOCX) [file pone.0352160.s002.docx]

# **S2 Power Decade and Frailty status (Women)**

| **Status** | **Decade** | **n = 2384** | **Mean** | **‘centile 05** | **‘centile 25** | **‘centile 50** | **‘centile**  **75** | **‘centile 95** | **Kurtosis / Skewness** |
| --- | --- | --- | --- | --- | --- | --- | --- | --- | --- |
| **Non-Frail** | 50 | 474 | 2.65 | 1.84 | 2.23 | 2.58 | 2.95 | 3.8 | 2.3 / 1.1 |
| **n= 1138** | 60 | 493 | 2.44 | 1.68 | 2.07 | 2.39 | 2.73 | 3.34 | 0.91 /0.68 |
|  | 70 | 143 | 2.31 | 1.66 | 1.95 | 2.23 | 2.57 | 3.36 | 1.69 / 1.15 |
|  | 80 | 28 | 2.02 | 1.35 | 1.75 | 1.99 | 2.23 | 2.9 | -0.16 / 0.55 |
| **Pre-Frail** | 50 | 205 | 2.4 | 1.67 | 1.99 | 2.34 | 2.74 | 3.46 | -0.05 / 0.62 |
| **n= 812** | 60 | 321 | 2.3 | 1.6 | 1.94 | 2.2 | 2.59 | 3.25 | 0.51 / 0.70 |
|  | 70 | 213 | 2.09 | 1.34 | 1.78 | 2.04 | 2.35 | 2.97 | 0.76 / 0.51 |
|  | 80 | 73 | 1.94 | 1.04 | 1.63 | 1.95 | 2.2 | 2.86 | 1.05 / 0.39 |
| **Frail** | 50 | 85 | 2.27 | 1.38 | 1.95 | 2.23 | 2.61 | 3.18 | 3.67 / 0.95 |
| **n= 434** | 60 | 151 | 1.99 | 1.22 | 1.65 | 1.92 | 2.31 | 2.88 | 0.69 / 0.56 |
|  | 70 | 130 | 1.95 | 1.2 | 1.61 | 1.88 | 2.27 | 2.94 | 0.48 / 0.70 |
|  | 80 | 68 | 1.86 | 1.02 | 1.49 | 1.8 | 2.13 | 2.92 | 2.0 / 0.86 |
