# Supplementary material for: The role of estimated muscle power from a sit-to-stand test in determining frailty in community-dwelling older adults
Source: PLoS One. 2026 Jul 2;21(7):e0352160. doi: 10.1371/journal.pone.0352160 (PMC13327205; doi:10.1371/journal.pone.0352160)
Supplement: S3 Table — (DOCX) [file pone.0352160.s003.docx]

# **S3 Table 3 Power (by Decade and Frailty status (Men)**

| **Status** | **Decade** | **N= 1,898** | **Mean** | **‘centile**  **5** | **‘centile 25** | **‘centile 50** | **‘centile**  **75** | **‘centile 95** | **Kurtosis / Skewness** |
| --- | --- | --- | --- | --- | --- | --- | --- | --- | --- |
| **Non-Frail** | 50 | 363 | 3.11 | 2.15 | 2.66 | 3.04 | 3.55 | 4.2 | 0.74 / 0.66 |
| **(n= 1,027)** | 60 | 458 | 3.01 | 2.02 | 2.51 | 2.92 | 3.37 | 4.16 | 3.62 / 1.2 |
|  | 70 | 192 | 2.87 | 1.99 | 2.41 | 2.77 | 3.28 | 4.07 | 1.15 / 0.76 |
|  | 80 | 14 | 2.9 | 1.99 | 2.34 | 2.95 | 3.26 | - | -0.09 / 0.48 |
| **Pre-Frail** | 50 | 125 | 2.9 | 2.03 | 2.48 | 2.87 | 3.28 | 4 | 1.15 / 0.73 |
| **(n= 638)** | 60 | 239 | 2.74 | 1.79 | 2.28 | 2.72 | 3.17 | 3.75 | 1.02 / 0.63 |
|  | 70 | 203 | 2.49 | 1.71 | 2.11 | 2.43 | 2.79 | 3.46 | 2.04 / 0.93 |
|  | 80 | 71 | 2.46 | 1.51 | 1.95 | 2.42 | 2.87 | 3.53 | -0.04 / 0.44 |
| **Frail** | 50 | 40 | 2.61 | 1.23 | 2.16 | 2.55 | 3.15 | 3.81 | 1.00 / 0.31 |
| **(n= 223)** | 60 | 73 | 2.54 | 1.67 | 2 | 2.47 | 3.02 | 3.68 | - 0.18 / 0.45 |
|  | 70 | 70 | 2.3 | 1.45 | 1.98 | 2.27 | 2.62 | 3.24 | -0.26 / 0.29 |
|  | 80 | 50 | 2.25 | 1.37 | 1.87 | 2.16 | 2.59 | 3.42 | 1.37 / 0.97 |
