# Supplementary material for: The role of estimated muscle power from a sit-to-stand test in determining frailty in community-dwelling older adults
Source: PLoS One. 2026 Jul 2;21(7):e0352160. doi: 10.1371/journal.pone.0352160 (PMC13327205; doi:10.1371/journal.pone.0352160)
Supplement: S4 Table — (DOCX) [file pone.0352160.s004.docx]

# **S4: Tabel 4**

| **Metric** | **TIME** | **POWER** |
| --- | --- | --- |
| -2 Log Likelihood | 1817.96 | 1822.52 |
| Cox & Snell R² | 0.32 | 0.31 |
| Nagelkerke R² | 0.48 | 0.48 |
| Omnibus χ² (df = 6) | 986.9, p<.001 | 982.3, p<.001 |
| Hosmer-Lemeshow Test   (p > .05) | χ²=12.67, p=.124 | χ²=12.95, p=.114 |
