# Supplementary material for: The role of estimated muscle power from a sit-to-stand test in determining frailty in community-dwelling older adults
Source: PLoS One. 2026 Jul 2;21(7):e0352160. doi: 10.1371/journal.pone.0352160 (PMC13327205; doi:10.1371/journal.pone.0352160)
Supplement: S5 Table — (DOCX) [file pone.0352160.s005.docx]

# **S5 Table 5 Correlations Between Frailty and Physical Measures**

| Variables | 1 | 2 | 3 | 4 | 5 |
| --- | --- | --- | --- | --- | --- |
| 1. Frailty Index | — |  |  |  |  |
| 2. TUG Time (s) | .45*** [0.43, 0.48] | — |  |  |  |
| 3. Grip Strength (kg) | –.36*** [–.39, –.33] | –.18*** [–.21, –.15] | — |  |  |
| 4. Muscle Power (watts) | –.37*** [–.39, –.34] | –.45*** [–.47, –.42] | .49*** [0.47, 0.51] | — |  |
| 5. Chair Stand Time (s) | .31*** [0.29, 0.34] | .48*** [0.46, 0.51] | –.18*** [–.21, –.15] | –.86*** [–.87, –.85] | — |

Note. Values are Spearman’s rho (ρ). Values in brackets are 95% confidence intervals. TUG = Timed Up and Go test. *p < .05, **p < .01, ***p < .001.
