# Supplementary material for: The role of estimated muscle power from a sit-to-stand test in determining frailty in community-dwelling older adults
Source: PLoS One. 2026 Jul 2;21(7):e0352160. doi: 10.1371/journal.pone.0352160 (PMC13327205; doi:10.1371/journal.pone.0352160)
Supplement: S6 File — (DOCX) [file pone.0352160.s006.docx]

# **S6 Frailty Index**

Sum of self-reported and physical tests

| Item No | Item | Recorded | Scored |
| --- | --- | --- | --- |
| 1 | Self-reported difficulty walking 100 metres | YES / NO | Yes = 1 |
| 2 | Self-reported difficulty rising from a chair after prolonged sitting | YES / NO | Yes = 1 |
| 3 | Self-reported difficulty climbing several sets of steps | YES / NO | Yes = 1 |
| 4 | Self-reported difficulty reaching above shoulder height | YES / NO | Yes = 1 |
| 5 | Self-reported difficulty pushing/pulling large objects | YES / NO | Yes = 1 |
| 6 | Self-reported difficulty lifting/carrying weights ≥10lb | YES / NO | Yes = 1 |
| 7 | Self-reported difficulty picking a coin up from a table | YES / NO | Yes = 1 |
| 8 | Self-rated general health | Likert scale: Excellent to Poor | Poor = 1  Fair = 0.5 |
| 9 | Self-rated day-to-day memory | Likert scale: Excellent to Poor | Poor = 1  Fair = 0.5 |
| 10 | Self-reported Urinary incontinence | YES / NO | Yes = 1 |
| 11 | Self-reported High blood pressure/hypertension | YES / NO | Yes = 1 |
| 12 | Self-reported Angina | YES / NO | Yes = 1 |
| 13 | Self-reported Heart attack | YES / NO | Yes = 1 |
| 14 | Self-reported Diabetes | YES / NO | Yes = 1 |
| 15 | Self-reported High cholesterol | YES / NO | Yes = 1 |
| 16 | Self-reported Abnormal heart rhythm | YES / NO | Yes = 1 |
| 17 | Polpharmacy | YES / NO | Yes =1 |
| 18 | Self-reported Unintentional Weight loss | YES / NO | Yes =1 |
| 19 | Self-reported osteoporosis | YES / NO | Yes = 1 |
| 20 | Self-reported Cancer diagnosis | YES / NO | Yes =1 |
| 21 | Low grip strength | Lowest 10 percentile of grip strength for sex | Yes = 1 |
| 22 | Slow walk speed | Lowest 10 percentile of Time up and go speed (m/sec) | Yes = 1 |

Index = Sum of 22 items / no items scored

Non Frail =  < 0.1

Pre-frail = 0.11-0.24

Frail ≥0.25
