# Supplementary material for: The role of estimated muscle power from a sit-to-stand test in determining frailty in community-dwelling older adults
Source: PLoS One. 2026 Jul 2;21(7):e0352160. doi: 10.1371/journal.pone.0352160 (PMC13327205; doi:10.1371/journal.pone.0352160)
Supplement: S7 File — (DOCX) [file pone.0352160.s007.docx]

**S7 Sensitivity Analysis**

A sensitivity analysis was conducted to assess whether removing of the item “Difficulty rising from a chair after prolonged sitting” materially affected the association between Frailty status and model predictors. Logistic regression using the original 22-item Frailty Index was compared with a model based on a 21-item index (excluding the chair rising item). Model performance and parameter estimates were highly consistent across analyses. The Nagelkerke R² increased only marginally from 0.472 to 0.494, and overall classification accuracy improved slightly from 86.0% to 87.4%. The direction, magnitude, and statistical significance of all predictors (grip strength, Power, timed up-and-go, sex, and BMI) remained unchanged. These results indicate that the findings are robust and not sensitive to the inclusion or exclusion of the “difficulty getting up from a chair” item.
