# Supplementary material for: The role of estimated muscle power from a sit-to-stand test in determining frailty in community-dwelling older adults
Source: PLoS One. 2026 Jul 2;21(7):e0352160. doi: 10.1371/journal.pone.0352160 (PMC13327205; doi:10.1371/journal.pone.0352160)
Supplement: Attachment — Submitted filename: Response to Reviewers 23 Feb.docx [file pone.0352160.s008.docx]

Discipline of Physiotherapy

Trinity Centre for Health Sciences

James St

Dublin 8

Ireland

18 Feb 2026

Re: PONE-D-25-61976

Dear Editor,

Thank you for your positive response to our submission PONE-D-25-61976.

In this letter, we address the points you and the reviewers raised.

Reviewer 2

Reviewer 2 provided a synopsis of the paper but did not request any specific changes nor recommend any alterations. We thank the reviewer for his/her time.

Reviewer 1

Reviewer 1 provided more specific commentary, which we have addressed in the revised text. We also thank the reviewer for his/her time.

“*The clarity and readability of the text could be enhanced.*

*Some sections, particularly in the Introduction and Discussion, contain long, complex sentences and repeated information that could be condensed.”*

The text throughout the revised submission has been reviewed, and improvements to grammar and syntax have been made as illustrated in the tracked changes document.

Where repeated information has been identified, this has been removed where appropriate. For example, line 235 pg 6 was removed.

The revised text contains the tracked changes throughout and has been analysed using Grammarly to improve the clarity of writing. The tracked changes document illustrates this.

*“The cross-sectional design of the study limits causal interpretations. Although this is acknowledged in the discussion, further emphasis on the implications for longitudinal or intervention studies would be beneficial.”*

We recognise that secondary data analysis limits causal inference. However, this study was based on only one wave of the TILDA study, which is a multi-wave longitudinal study. We will conduct follow-up studies in subsequent waves to validate the findings of this study, notably to examine whether the cut-offs classified persons who were non-frail or pre-frail at Wave 3 who developed frailty in subsequent waves. Whilst we focused on a binary outcome of Frail vs Non Frail, we will include Prefrail in future work. We elected to focus on the binary outcome to test whether the estimated muscle power was a suitable tool. See lines 393

Editor comments

Editor comments were very welcome, and we note and respond to the points raised:

*“The use of a large, nationally representative sample is a notable strength, enhancing external validity within community-dwelling older adults. Nevertheless, the exclusion of participants unable to complete the five-times sit-to-stand test may introduce selection bias, potentially leading to an underrepresentation of individuals with more severe frailty or functional impairment. Additionally, the age distribution is skewed toward younger older adults, with over 70% of participants under 70 years of age, which may limit the generalizability of the proposed cut-off values to more advanced age groups or institutionalized populations.”*

We acknowledge that the use of retrospective data is a limitation in this study. As the purpose of the paper was to explore the role of estimates of muscle power, we are limited to data that allow muscle power to be estimated over directly measured power. The rationale for using muscle power as a marker of functional ability is to assist health care professionals in identifying those persons who are currently non-frail or pre-frail, subject to different Frailty classifications and thus intervene to prevent frailty developing. Within this dataset, where most of the participants are under 70, there are approximately 40% between ages 60 – 70, so many of these individuals will be in the over-70 category in subsequent waves of the TILDA study. This will enable us to follow them across multiple time points (TILDA Wave 5 and 6) to assess the validity of this study.

(pg 9, LINE 337 - Whilst this study limited itself to a binary approach (frail vs non-frail), the role of muscle power in the pre-frail group would be of interest to follow over time, to determine whether pre-frail persons develop frailty or can reverse to non-frail.

*“ Frailty is operationalized using a deficit accumulation Frailty Index based on 22 items, which is consistent with established methodology and well justified. However, the application of fixed cut-off points and the subsequent dichotomization of frailty status for regression analyses result in a loss of information, particularly for individuals classified as pre-frail.”*

The cutoff points were selected based on published literature, and the purpose was to assess whether the muscle power could identify frailty. We recognise that prefrail individuals were not included in this analysis, but this is the next stage of our work, in which we will follow up those considered prefrail at Wave 3 to see whether they developed additional impairments at subsequent Waves. The concept of ‘prefrail’ has not been widely assessed as frailty can be reversed in some cases. Thus, some individuals classed as being prefrail may not progress to frailty or may be deemed non-frail. This is often due to the assessment tool used for frailty. We chose one commonly used approach that the data available to us could provide.

*“Estimated muscle power is derived using a previously validated equation based on the sit-to-stand test, incorporating anthropometric measures and chair height. This approach is pragmatic and clinically feasible, but it remains an indirect estimate based on a submaximal functional task. However, the reported AUC values indicate moderate rather than high discrimination, and this should be more cautiously framed in the interpretation of diagnostic accuracy. Furthermore, potential interaction effects, particularly between sex and muscle power, are not clearly explored and may merit additional analysis.”*

We concur with the comments and have amended the text to reflect a more moderate interpretation of the AUC. (Line 276, pg 8)

The additional supporting reference (Mandrekar, J. N., 2010) indicates that AUC between 0.7 and 0.8 is considered acceptable.

In the results section, on pg 8, line 269, commentary on the additional regression analysis conducted with an additional variable (Gender X Power) was included. This was non-significant.

“However, the interaction between gender and muscle power was not significant (OR 0.82 CI 0.52-1.28, p=0.38), indicating that the protective effect of muscle power did not differ significantly between males and females.”

As the interaction was non-significant, we have not pursued this in the discussion

However, to illustrate the impact of power on the different genders, we have reflected this in an updated Figure 2, which separates males and females and plots predicted frailty against estimated Watts per kg.

We hope that the amendments to the main text address all the points raised and look forward to seeing this published in PLOS one

Yours

Cillin Condon
