# Supplementary material for: The role of estimated muscle power from a sit-to-stand test in determining frailty in community-dwelling older adults
Source: PLoS One. 2026 Jul 2;21(7):e0352160. doi: 10.1371/journal.pone.0352160 (PMC13327205; doi:10.1371/journal.pone.0352160)
Supplement: Attachment — Submitted filename: Response to Reviewers PLOS v2.docx [file pone.0352160.s009.docx]

17 May 2026

Dear PLOS Editor,

Thank you and the reviewers for your attention to this paper.

As requested, we have addressed the minor clarifications and adjustments required. We hope these meet the requirements for publication. I have outlined how and where we have addressed the comments from the reviewers.

Additional reference:

One new reference was added to the list to support point no 1 raised by the reviewer. (No 32 Seligman et al, 2026). No references were removed or identified as being retracted.

Comments from Reviewer:

1	First, the definition of frailty categories would benefit from clarification. As currently reported, individuals with a Frailty Index of exactly 0.25 do not appear to fall into any category, as pre-frail is defined as 0.11–0.24 and frail as >0.25. It would be helpful to revise this threshold (e.g., ≥0.25) and confirm whether any participants were affected by this definition.

Adjustment

In Methods, we have corrected the classification for Frailty to include the ≥ symbol rather than > . We further confirm that the distribution of people by Frailty Status did not change and that the ≥ should have been included in the originally submitted draft. In addition, we have clarified that the Index includes two physical parameters. This did not affect the distribution.

Comments from Reviewer:

2. The exclusion of pre-frail individuals from the main regression analyses could be more explicitly justified. While the rationale is briefly mentioned, a short clarification in the Methods or Discussion would help readers better understand this choice, particularly given the clinical relevance of the pre-frail group.

Adjustment

Under Methods, the following has been added.

“The prefrail group was not included at this time, as the discriminatory ability of the estimated muscle power has not been fully established; including the prefrail group would be premature until this has been assessed.

In the Discussion section, this is addressed under the amended sentence.

“Whilst this study limited itself to a binary approach (frail vs non-frail), the role of muscle power in the pre-frail group would be of interest to follow over time, to determine whether pre-frail persons develop frailty or can reverse to non-frail status, as this may confer greater health benefits for the ageing population. Longitudinal studies, such as the TILDA study, can assess this over time.”

Comments from Reviewer:

3. The interpretation of diagnostic performance could be slightly moderated for consistency with the reported results. The AUC values (~0.74–0.75) suggest moderate discrimination, and aligning the wording in the Abstract and Discussion with this level of performance would improve clarity.

Adjustment

We have amended the Abstract to reflect the comments.
Changing word ‘high’ to ‘moderate’ agreement and clarifying that estimating muscle power does not replace other methods, but can complement them

“Muscle power estimates showed moderate agreement with frailty status, with sensitivity and specificity comparable to those of the 5xSTS. Muscle power less than 2.5 Watt·kg⁻¹ in males and 2.08 Watt·kg⁻¹ in females was associated with increased frailty risk, consistent with other studies. Overall power estimation showed a predictive performance similar to that of traditional assessments such as Timed Up and Go, supporting its utility in clinical and community settings.

Conclusion. Estimated muscle power derived from the 5xSTS test is a practical, reliable tool for early identification of frailty among older adults. Its accessibility and predictive validity suggest it could complement existing clinical assessments, but not replace them.”

Comments from Reviewer:

4. There appears to be some ambiguity in the presentation and interpretation of the muscle power cut-off values. In parts of the manuscript, lower values are associated with increased frailty risk, whereas in the ROC analysis thresholds are expressed using “≥” notation. A brief clarification of the directionality and clinical interpretation of these cut-offs would help avoid potential confusion for readers.

Adjustment

In the Results section, the paragraph related to ROC has been updated and the notation corrected to ( > ).

“To explore the proposition of cutoffs for defining frailty, described by Alcazar et al.(Alcazar et al., 2021), a two-way scatter plot was created to show predicted frailty against the continuous power variable (Figure 2) for both males and females. The figure shows a decline in predicted frailty with increasing muscle power. The optimal cutoff based on the Youden Index, where the chance of being frail drops significantly, for men, was **> 2.50** **Watt.Kg-^1^**, yielding a sensitivity of **77.2%** and a specificity of **61.4%**. For women, the optimal cutoff was **>2.07 Watt.Kg-^1^**, with a sensitivity of **77.6%** and a specificity of **60.4%**.
A receiver operating characteristic (ROC) analysis was conducted to evaluate the ability of **power** to identify frailty / non-frailty classification.”

Comments from Reviewer:

5. In addition, while the coding of the dependent variable (non-frail = 1, frail = 0) is appropriate, a short clarification on how to interpret the resulting odds ratios (i.e., in terms of likelihood of being non-frail rather than risk of frailty) would improve clarity.

Adjustment

In the discussion section, we have amended the section to clarify the results of the Odds Ratio and the direction referred to by the reviewer.

“Our study reported an odds ratio of 1.83 for muscle power predicting frailty, which, when inverted (1/1.83 =55%), means that for each unit increase in power (1 Watt·kg⁻¹), the chance of being frail is reduced by 55%. Although it should be noted that changes of 0.1 -0.2 are more likely to occur.”

Comments from Reviewer:

6. Finally, as estimated muscle power is derived directly from the 5xSTS test, it would be useful to more explicitly acknowledge that the comparison between muscle power and 5xSTS time reflects closely related measures rather than fully independent constructs.

Adjustment

Our Limitations section has been amended to include the following:

“The power presented here is still an estimate, based on individuals' physical performance, using indirect equations rather than a direct measure of the muscles involved in the sit-to-stand action. These are closely related (supplemental data, r = -.82, increased chair stand times are highly but negatively correlated with estimated power) and not fully independent constructs.”

We are grateful to the reviewers and to you for your time and comments in bringing the paper to this stage and look forward to seeing it published.

Kind regards

Cillin Condon

Regards

Cillin Condon
