# Supplementary material for: The role of estimated muscle power from a sit-to-stand test in determining frailty in community-dwelling older adults
Source: PLoS One. 2026 Jul 2;21(7):e0352160. doi: 10.1371/journal.pone.0352160 (PMC13327205; doi:10.1371/journal.pone.0352160)
Supplement: Peer review history

# Peer review: Decision Letter 0

**DOI:** 10.1371/journal.pone.0352160.r001

**Authors:** Francesco Curcio

3 Feb 2026

PONE-D-25-61976

The Role of Estimated Muscle Power from a Sit-to-Stand test in determining Frailty in Community-Dwelling Older Adults

PLOS One

Dear Dr. Condon,

Thank you for submitting your manuscript to PLOS ONE. After careful consideration, we feel that it has merit but does not fully meet PLOS ONE’s publication criteria as it currently stands. Therefore, we invite you to submit a revised version of the manuscript that addresses the points raised during the review process.

Please submit your revised manuscript by Mar 20 2026 11:59PM. If you will need more time than this to complete your revisions, please reply to this message or contact the journal office at plosone@plos.org. When you're ready to submit your revision, log on to https://www.editorialmanager.com/pone/ and select the 'Submissions Needing Revision' folder to locate your manuscript file.

Please include the following items when submitting your revised manuscript:

- A letter that responds to each point raised by the academic editor and reviewer(s). You should upload this letter as a separate file labeled 'Response to Reviewers'.
- A marked-up copy of your manuscript that highlights changes made to the original version. You should upload this as a separate file labeled 'Revised Manuscript with Track Changes'.
- An unmarked version of your revised paper without tracked changes. You should upload this as a separate file labeled 'Manuscript'.

-->If you would like to make changes to your financial disclosure, please include your updated statement in your cover letter. Guidelines for resubmitting your figure files are available below the reviewer comments at the end of this letter.

If applicable, we recommend that you deposit your laboratory protocols in protocols.io to enhance the reproducibility of your results. Protocols.io assigns your protocol its own identifier (DOI) so that it can be cited independently in the future. For instructions see: https://journals.plos.org/plosone/s/submission-guidelines#loc-laboratory-protocols. Additionally, PLOS ONE offers an option for publishing peer-reviewed Lab Protocol articles, which describe protocols hosted on protocols.io. Read more information on sharing protocols at https://plos.org/protocols?utm_medium=editorial-email&utm_source=authorletters&utm_campaign=protocols.

We look forward to receiving your revised manuscript.

Kind regards,

Francesco Curcio, M.D., Ph.D.

Academic Editor

PLOS One

Journal Requirements:

When submitting your revision, we need you to address these additional requirements.

1. Please ensure that your manuscript meets PLOS ONE's style requirements, including those for file naming. The PLOS ONE style templates can be found at

https://journals.plos.org/plosone/s/file?id=wjVg/PLOSOne_formatting_sample_main_body.pdf and

https://journals.plos.org/plosone/s/file?id=ba62/PLOSOne_formatting_sample_title_authors_affiliations.pdf

2. When completing the data availability statement of the submission form, you indicated that you will make your data available on acceptance. We strongly recommend all authors decide on a data sharing plan before acceptance, as the process can be lengthy and hold up publication timelines. Please note that, though access restrictions are acceptable now, your entire data will need to be made freely accessible if your manuscript is accepted for publication. This policy applies to all data except where public deposition would breach compliance with the protocol approved by your research ethics board. If you are unable to adhere to our open data policy, please kindly revise your statement to explain your reasoning and we will seek the editor's input on an exemption. Please be assured that, once you have provided your new statement, the assessment of your exemption will not hold up the peer review process.

3. Please include captions for your Supporting Information files at the end of your manuscript, and update any in-text citations to match accordingly. Please see our Supporting Information guidelines for more information: http://journals.plos.org/plosone/s/supporting-information.

4. If the reviewer comments include a recommendation to cite specific previously published works, please review and evaluate these publications to determine whether they are relevant and should be cited. There is no requirement to cite these works unless the editor has indicated otherwise.

5. Please review your reference list to ensure that it is complete and correct. If you have cited papers that have been retracted, please include the rationale for doing so in the manuscript text, or remove these references and replace them with relevant current references. Any changes to the reference list should be mentioned in the rebuttal letter that accompanies your revised manuscript. If you need to cite a retracted article, indicate the article’s retracted status in the References list and also include a citation and full reference for the retraction notice.

**Additional Editor Comments:**

The use of a large, nationally representative sample is a notable strength, enhancing external validity within community-dwelling older adults. Nevertheless, the exclusion of participants unable to complete the five-times sit-to-stand test may introduce selection bias, potentially leading to an underrepresentation of individuals with more severe frailty or functional impairment. Additionally, the age distribution is skewed toward younger older adults, with over 70% of participants under 70 years of age, which may limit the generalizability of the proposed cut-off values to more advanced age groups or institutionalized populations.

Frailty is operationalized using a deficit accumulation Frailty Index based on 22 items, which is consistent with established methodology and well justified. However, the application of fixed cut-off points and the subsequent dichotomization of frailty status for regression analyses result in a loss of information, particularly for individuals classified as pre-frail.

Estimated muscle power is derived using a previously validated equation based on the sit-to-stand test, incorporating anthropometric measures and chair height. This approach is pragmatic and clinically feasible, but it remains an indirect estimate based on a submaximal functional task.

However, the reported AUC values indicate moderate rather than high discrimination, and this should be more cautiously framed in the interpretation of diagnostic accuracy. Furthermore, potential interaction effects, particularly between sex and muscle power, are not clearly explored and may merit additional analysis.

[Note: HTML markup is below. Please do not edit.]

Reviewers' comments:

Reviewer's Responses to Questions

**Comments to the Author**

1. Is the manuscript technically sound, and do the data support the conclusions?

The manuscript must describe a technically sound piece of scientific research with data that supports the conclusions. Experiments must have been conducted rigorously, with appropriate controls, replication, and sample sizes. The conclusions must be drawn appropriately based on the data presented.

Reviewer #1: Yes

Reviewer #2: Yes

**********

2. Has the statistical analysis been performed appropriately and rigorously?

Reviewer #1: Yes

Reviewer #2: Yes

**********

3. Have the authors made all data underlying the findings in their manuscript fully available?

The PLOS Data policy requires authors to make all data underlying the findings described in their manuscript fully available without restriction, with rare exception (please refer to the Data Availability Statement in the manuscript PDF file). The data should be provided as part of the manuscript or its supporting information, or deposited to a public repository. For example, in addition to summary statistics, the data points behind means, medians and variance measures should be available. If there are restrictions on publicly sharing data—e.g. participant privacy or use of data from a third party—those must be specified.

Reviewer #1: Yes

Reviewer #2: Yes

**********

4. Is the manuscript presented in an intelligible fashion and written in standard English?

PLOS ONE does not copyedit accepted manuscripts, so the language in submitted articles must be clear, correct, and unambiguous. Any typographical or grammatical errors should be corrected at revision, so please note any specific errors here.

Reviewer #1: No

Reviewer #2: Yes

**********

5. Review Comments to the Author

Please use the space provided to explain your answers to the questions above. You may also include additional comments for the author, including concerns about dual publication, research ethics, or publication ethics. (Please upload your review as an attachment if it exceeds 20,000 characters)

Reviewer #1: This manuscript explores the utility of estimating lower limb muscle power from the 5xSTS test as a predictor of frailty in older Irish adults. Overall, the study addresses an important and clinically relevant question. The authors clearly present their research objectives, distinguishing between the primary aim (estimating lower limb power) and the secondary aim (examining its predictive capacity for frailty). Despite these strengths, several limitations and areas for improvement are apparent:

The clarity and readability of the text could be enhanced. Some sections, particularly in the Introduction and Discussion, contain long, complex sentences and repeated information that could be condensed.

The cross-sectional design of the study limits causal interpretations. Although this is acknowledged in the discussion, further emphasis on the implications for longitudinal or intervention studies would be beneficial.

Reviewer #2: This study explored the usefulness of estimated lower limb muscle power, derived from the 5 times sit-to-stand (5xSTS) test, at identifying frailty among community-dwelling older adults in Ireland. Data derived from The Irish Longitudinal Study on Ageing focusing on adults aged 50 years and older. Analysis was performed on 4,295 subjects. The results reveal a decline in muscle power with advancing age, more pronounced in females and frail individuals. Muscle power estimates demonstrated high agreement with frailty status, with comparable sensitivity and specificity to the 5xSTS. Muscle power less than 2.5 Watt·kg−1 for males and 2.08 Watt·kg−1 for females was associated with increased frailty risk, aligning with other studies. Overall power estimation showed similar predictive performance to traditional assessments, supporting its utility in clinical and community settings.

I found the manuscript of interest. The topic is relevant. The methodology is correct and the analysis is performed on a large number of subjects. The clinical significance is important for geriatrician,

**********

6. PLOS authors have the option to publish the peer review history of their article (what does this mean?). If published, this will include your full peer review and any attached files.

If you choose “no”, your identity will remain anonymous but your review may still be made public.

**Do you want your identity to be public for this peer review?**  For information about this choice, including consent withdrawal, please see our Privacy Policy.

Reviewer #1: No

Reviewer #2: No

**********

[NOTE: If reviewer comments were submitted as an attachment file, they will be attached to this email and accessible via the submission site. Please log into your account, locate the manuscript record, and check for the action link "View Attachments". If this link does not appear, there are no attachment files.]

To ensure your figures meet our technical requirements, please review our figure guidelines: https://journals.plos.org/plosone/s/figures

You may also use PLOS’s free figure tool, NAAS, to help you prepare publication quality figures: https://journals.plos.org/plosone/s/figures#loc-tools-for-figure-preparation.

NAAS will assess whether your figures meet our technical requirements by comparing each figure against our figure specifications.

# Author comment: Author response to Decision Letter 1

**DOI:** 10.1371/journal.pone.0352160.r002

5 Mar 2026

Re: PONE-D-25-61976

Dear Editor,

Thank you for your positive response to our submission PONE-D-25-61976.

In this letter, we address the points you and the reviewers raised.

Reviewer 2

Reviewer 2 provided a synopsis of the paper but did not request any specific changes nor recommend any alterations. We thank the reviewer for his/her time.

Reviewer 1

Reviewer 1 provided more specific commentary, which we have addressed in the revised text. We also thank the reviewer for his/her time.

“The clarity and readability of the text could be enhanced.

Some sections, particularly in the Introduction and Discussion, contain long, complex sentences and repeated information that could be condensed.”

The text throughout the revised submission has been reviewed, and improvements to grammar and syntax have been made as illustrated in the tracked changes document.

Where repeated information has been identified, this has been removed where appropriate. For example, line 235 pg 6 was removed.

The revised text contains the tracked changes throughout and has been analysed using Grammarly to improve the clarity of writing. The tracked changes document illustrates this.

“The cross-sectional design of the study limits causal interpretations. Although this is acknowledged in the discussion, further emphasis on the implications for longitudinal or intervention studies would be beneficial.”

We recognise that secondary data analysis limits causal inference. However, this study was based on only one wave of the TILDA study, which is a multi-wave longitudinal study. We will conduct follow-up studies in subsequent waves to validate the findings of this study, notably to examine whether the cut-offs classified persons who were non-frail or pre-frail at Wave 3 who developed frailty in subsequent waves. Whilst we focused on a binary outcome of Frail vs Non Frail, we will include Prefrail in future work. We elected to focus on the binary outcome to test whether the estimated muscle power was a suitable tool. See lines 393

Editor comments

Editor comments were very welcome, and we note and respond to the points raised:

“The use of a large, nationally representative sample is a notable strength, enhancing external validity within community-dwelling older adults. Nevertheless, the exclusion of participants unable to complete the five-times sit-to-stand test may introduce selection bias, potentially leading to an underrepresentation of individuals with more severe frailty or functional impairment. Additionally, the age distribution is skewed toward younger older adults, with over 70% of participants under 70 years of age, which may limit the generalizability of the proposed cut-off values to more advanced age groups or institutionalized populations.”

We acknowledge that the use of retrospective data is a limitation in this study. As the purpose of the paper was to explore the role of estimates of muscle power, we are limited to data that allow muscle power to be estimated over directly measured power. The rationale for using muscle power as a marker of functional ability is to assist health care professionals in identifying those persons who are currently non-frail or pre-frail, subject to different Frailty classifications and thus intervene to prevent frailty developing. Within this dataset, where most of the participants are under 70, there are approximately 40% between ages 60 – 70, so many of these individuals will be in the over-70 category in subsequent waves of the TILDA study. This will enable us to follow them across multiple time points (TILDA Wave 5 and 6) to assess the validity of this study.

(pg 9, LINE 337 - Whilst this study limited itself to a binary approach (frail vs non-frail), the role of muscle power in the pre-frail group would be of interest to follow over time, to determine whether pre-frail persons develop frailty or can reverse to non-frail.

“ Frailty is operationalized using a deficit accumulation Frailty Index based on 22 items, which is consistent with established methodology and well justified. However, the application of fixed cut-off points and the subsequent dichotomization of frailty status for regression analyses result in a loss of information, particularly for individuals classified as pre-frail.”

The cutoff points were selected based on published literature, and the purpose was to assess whether the muscle power could identify frailty. We recognise that prefrail individuals were not included in this analysis, but this is the next stage of our work, in which we will follow up those considered prefrail at Wave 3 to see whether they developed additional impairments at subsequent Waves. The concept of ‘prefrail’ has not been widely assessed as frailty can be reversed in some cases. Thus, some individuals classed as being prefrail may not progress to frailty or may be deemed non-frail. This is often due to the assessment tool used for frailty. We chose one commonly used approach that the data available to us could provide.

“Estimated muscle power is derived using a previously validated equation based on the sit-to-stand test, incorporating anthropometric measures and chair height. This approach is pragmatic and clinically feasible, but it remains an indirect estimate based on a submaximal functional task. However, the reported AUC values indicate moderate rather than high discrimination, and this should be more cautiously framed in the interpretation of diagnostic accuracy. Furthermore, potential interaction effects, particularly between sex and muscle power, are not clearly explored and may merit additional analysis.”

We concur with the comments and have amended the text to reflect a more moderate interpretation of the AUC. (Line 276, pg 8)

The additional supporting reference (Mandrekar, J. N., 2010) indicates that AUC between 0.7 and 0.8 is considered acceptable.

In the results section, on pg 8, line 269, commentary on the additional regression analysis conducted with an additional variable (Gender X Power) was included. This was non-significant.

“However, the interaction between gender and muscle power was not significant (OR 0.82 CI 0.52-1.28, p=0.38), indicating that the protective effect of muscle power did not differ significantly between males and females.”

As the interaction was non-significant, we have not pursued this in the discussion

However, to illustrate the impact of power on the different genders, we have reflected this in an updated Figure 2, which separates males and females and plots predicted frailty against estimated Watts per kg.

We hope that the amendments to the main text address all the points raised.

Yours

Cillin Condon

Attachment Submitted filename: Response to Reviewers 23 Feb.docx

# Peer review: Decision Letter 1

**DOI:** 10.1371/journal.pone.0352160.r003

**Authors:** Francesco Curcio, Francesco Curcio

4 May 2026

PONE-D-25-61976R1

The Role of Estimated Muscle Power from a Sit-to-Stand test in determining Frailty in Community-Dwelling Older Adults

PLOS One

Dear Dr. Condon,

Thank you for submitting your manuscript to PLOS ONE. After careful consideration, we feel that it has merit but does not fully meet PLOS ONE’s publication criteria as it currently stands. Therefore, we invite you to submit a revised version of the manuscript that addresses the points raised during the review process.

- **Please address minor revisions, focusing on clarification of frailty categorization, cut-off interpretation, and model specification. No additional analyses are required, but improved clarity in reporting and interpretation will strengthen the manuscript.**

-->Please submit your revised manuscript by Jun 18 2026 11:59PM. If you will need more time than this to complete your revisions, please reply to this message or contact the journal office at plosone@plos.org. When you're ready to submit your revision, log on to https://www.editorialmanager.com/pone/ and select the 'Submissions Needing Revision' folder to locate your manuscript file.

Please include the following items when submitting your revised manuscript:

- A letter that responds to each point raised by the academic editor and reviewer(s). You should upload this letter as a separate file labeled 'Response to Reviewers'.
- A marked-up copy of your manuscript that highlights changes made to the original version. You should upload this as a separate file labeled 'Revised Manuscript with Track Changes'.
- An unmarked version of your revised paper without tracked changes. You should upload this as a separate file labeled 'Manuscript'.

If you would like to make changes to your financial disclosure, please include your updated statement in your cover letter. Guidelines for resubmitting your figure files are available below the reviewer comments at the end of this letter.

If applicable, we recommend that you deposit your laboratory protocols in protocols.io to enhance the reproducibility of your results. Protocols.io assigns your protocol its own identifier (DOI) so that it can be cited independently in the future. For instructions see: https://journals.plos.org/plosone/s/submission-guidelines#loc-laboratory-protocols. Additionally, PLOS ONE offers an option for publishing peer-reviewed Lab Protocol articles, which describe protocols hosted on protocols.io. Read more information on sharing protocols at https://plos.org/protocols?utm_medium=editorial-email&utm_source=authorletters&utm_campaign=protocols.

As the corresponding author, your ORCID iD is verified in the submission system and will appear in the published article. PLOS supports the use of ORCID, and we encourage all coauthors to register for an ORCID iD and use it as well. Please encourage your coauthors to verify their ORCID iD within the submission system before final acceptance, as unverified ORCID iDs will not appear in the published article. *Only*  the individual author can complete the verification step; PLOS staff *cannot*  verify ORCID iDs on behalf of authors.

We look forward to receiving your revised manuscript.

Kind regards,

Francesco Curcio, M.D., Ph.D.

Academic Editor

PLOS One

**Journal Requirements:**

1. If the reviewer comments include a recommendation to cite specific previously published works, please review and evaluate these publications to determine whether they are relevant and should be cited. There is no requirement to cite these works unless the editor has indicated otherwise.

2. Please review your reference list to ensure that it is complete and correct. If you have cited papers that have been retracted, please include the rationale for doing so in the manuscript text, or remove these references and replace them with relevant current references. Any changes to the reference list should be mentioned in the rebuttal letter that accompanies your revised manuscript. If you need to cite a retracted article, indicate the article’s retracted status in the References list and also include a citation and full reference for the retraction notice.

**Additional Editor Comments:**

In line with the reviewers’ positive evaluations, I agree that the manuscript addresses a relevant and timely topic and is suitable for publication. The authors have made a clear effort to revise the manuscript and improve its clarity. Before proceeding further, however, I would appreciate a few additional clarifications and minor adjustments to strengthen the methodological transparency and overall interpretation of the findings.

First, the definition of frailty categories would benefit from clarification. As currently reported, individuals with a Frailty Index of exactly 0.25 do not appear to fall into any category, as pre-frail is defined as 0.11–0.24 and frail as >0.25. It would be helpful to revise this threshold (e.g., ≥0.25) and confirm whether any participants were affected by this definition.

Second, the exclusion of pre-frail individuals from the main regression analyses could be more explicitly justified. While the rationale is briefly mentioned, a short clarification in the Methods or Discussion would help readers better understand this choice, particularly given the clinical relevance of the pre-frail group.

Third, the interpretation of diagnostic performance could be slightly moderated for consistency with the reported results. The AUC values (~0.74–0.75) suggest moderate discrimination, and aligning the wording in the Abstract and Discussion with this level of performance would improve clarity.

There appears to be some ambiguity in the presentation and interpretation of the muscle power cut-off values. In parts of the manuscript, lower values are associated with increased frailty risk, whereas in the ROC analysis thresholds are expressed using “≥” notation. A brief clarification of the directionality and clinical interpretation of these cut-offs would help avoid potential confusion for readers. In addition, while the coding of the dependent variable (non-frail = 1, frail = 0) is appropriate, a short clarification on how to interpret the resulting odds ratios (i.e., in terms of likelihood of being non-frail rather than risk of frailty) would improve clarity.

Finally, as estimated muscle power is derived directly from the 5xSTS test, it would be useful to more explicitly acknowledge that the comparison between muscle power and 5xSTS time reflects closely related measures rather than fully independent constructs.

[Note: HTML markup is below. Please do not edit.]

Reviewers' comments:

Reviewer's Responses to Questions

**Comments to the Author**

1. If the authors have adequately addressed your comments raised in a previous round of review and you feel that this manuscript is now acceptable for publication, you may indicate that here to bypass the “Comments to the Author” section, enter your conflict of interest statement in the “Confidential to Editor” section, and submit your "Accept" recommendation.

Reviewer #2: All comments have been addressed

**********

2. Is the manuscript technically sound, and do the data support the conclusions?

The manuscript must describe a technically sound piece of scientific research with data that supports the conclusions. Experiments must have been conducted rigorously, with appropriate controls, replication, and sample sizes. The conclusions must be drawn appropriately based on the data presented.

Reviewer #2: Yes

**********

3. Has the statistical analysis been performed appropriately and rigorously?

Reviewer #2: Yes

**********

4. Have the authors made all data underlying the findings in their manuscript fully available?

The PLOS Data policy requires authors to make all data underlying the findings described in their manuscript fully available without restriction, with rare exception (please refer to the Data Availability Statement in the manuscript PDF file). The data should be provided as part of the manuscript or its supporting information, or deposited to a public repository. For example, in addition to summary statistics, the data points behind means, medians and variance measures should be available. If there are restrictions on publicly sharing data—e.g. participant privacy or use of data from a third party—those must be specified.

Reviewer #2: Yes

**********

5. Is the manuscript presented in an intelligible fashion and written in standard English?

PLOS ONE does not copyedit accepted manuscripts, so the language in submitted articles must be clear, correct, and unambiguous. Any typographical or grammatical errors should be corrected at revision, so please note any specific errors here.

Reviewer #2: Yes

**********

6. Review Comments to the Author

Please use the space provided to explain your answers to the questions above. You may also include additional comments for the author, including concerns about dual publication, research ethics, or publication ethics. (Please upload your review as an attachment if it exceeds 20,000 characters)

**Reviewer #2:**  The manuscript is improved and I have no more suggestions to improve the paper that is acceptable for publication in the present form

**********

7. PLOS authors have the option to publish the peer review history of their article (what does this mean?). If published, this will include your full peer review and any attached files.

If you choose “no”, your identity will remain anonymous but your review may still be made public.

**Do you want your identity to be public for this peer review?**  For information about this choice, including consent withdrawal, please see our Privacy Policy.

Reviewer #2: No

**********

[NOTE: If reviewer comments were submitted as an attachment file, they will be attached to this email and accessible via the submission site. Please log into your account, locate the manuscript record, and check for the action link "View Attachments". If this link does not appear, there are no attachment files.]

To ensure your figures meet our technical requirements, please review our figure guidelines: https://journals.plos.org/plosone/s/figures

You may also use PLOS’s free figure tool, NAAS, to help you prepare publication quality figures: https://journals.plos.org/plosone/s/figures#loc-tools-for-figure-preparation.

NAAS will assess whether your figures meet our technical requirements by comparing each figure against our figure specifications.

# Author comment: Author response to Decision Letter 2

**DOI:** 10.1371/journal.pone.0352160.r004

21 May 2026

17 May 2026

Dear PLOS Editor,

Thank you and the reviewers for your attention to this paper.

As requested, we have addressed the minor clarifications and adjustments required. We hope these meet the requirements for publication. I have outlined how and where we have addressed the comments from the reviewers.

Additional reference:

One new reference was added to the list to support point no 1 raised by the reviewer. (No 32 Seligman et al, 2026). No references were removed or identified as being retracted.

Comments from Reviewer:

1 First, the definition of frailty categories would benefit from clarification. As currently reported, individuals with a Frailty Index of exactly 0.25 do not appear to fall into any category, as pre-frail is defined as 0.11–0.24 and frail as >0.25. It would be helpful to revise this threshold (e.g., ≥0.25) and confirm whether any participants were affected by this definition.

Adjustment

In Methods, we have corrected the classification for Frailty to include the ≥ symbol rather than > . We further confirm that the distribution of people by Frailty Status did not change and that the ≥ should have been included in the originally submitted draft. In addition, we have clarified that the Index includes two physical parameters. This did not affect the distribution.

Comments from Reviewer:

2. The exclusion of pre-frail individuals from the main regression analyses could be more explicitly justified. While the rationale is briefly mentioned, a short clarification in the Methods or Discussion would help readers better understand this choice, particularly given the clinical relevance of the pre-frail group.

Adjustment

Under Methods, the following has been added.

“The prefrail group was not included at this time, as the discriminatory ability of the estimated muscle power has not been fully established; including the prefrail group would be premature until this has been assessed.

In the Discussion section, this is addressed under the amended sentence.

“Whilst this study limited itself to a binary approach (frail vs non-frail), the role of muscle power in the pre-frail group would be of interest to follow over time, to determine whether pre-frail persons develop frailty or can reverse to non-frail status, as this may confer greater health benefits for the ageing population. Longitudinal studies, such as the TILDA study, can assess this over time.”

Comments from Reviewer:

3. The interpretation of diagnostic performance could be slightly moderated for consistency with the reported results. The AUC values (~0.74–0.75) suggest moderate discrimination, and aligning the wording in the Abstract and Discussion with this level of performance would improve clarity.

Adjustment

We have amended the Abstract to reflect the comments.

Changing word ‘high’ to ‘moderate’ agreement and clarifying that estimating muscle power does not replace other methods, but can complement them

“Muscle power estimates showed moderate agreement with frailty status, with sensitivity and specificity comparable to those of the 5xSTS. Muscle power less than 2.5 Watt·kg⁻¹ in males and 2.08 Watt·kg⁻¹ in females was associated with increased frailty risk, consistent with other studies. Overall power estimation showed a predictive performance similar to that of traditional assessments such as Timed Up and Go, supporting its utility in clinical and community settings.

Conclusion. Estimated muscle power derived from the 5xSTS test is a practical, reliable tool for early identification of frailty among older adults. Its accessibility and predictive validity suggest it could complement existing clinical assessments, but not replace them.”

Comments from Reviewer:

4. There appears to be some ambiguity in the presentation and interpretation of the muscle power cut-off values. In parts of the manuscript, lower values are associated with increased frailty risk, whereas in the ROC analysis thresholds are expressed using “≥” notation. A brief clarification of the directionality and clinical interpretation of these cut-offs would help avoid potential confusion for readers.

Adjustment

In the Results section, the paragraph related to ROC has been updated and the notation corrected to ( > ).

“To explore the proposition of cutoffs for defining frailty, described by Alcazar et al.(Alcazar et al., 2021), a two-way scatter plot was created to show predicted frailty against the continuous power variable (Figure 2) for both males and females. The figure shows a decline in predicted frailty with increasing muscle power. The optimal cutoff based on the Youden Index, where the chance of being frail drops significantly, for men, was > 2.50 Watt.Kg-1, yielding a sensitivity of 77.2% and a specificity of 61.4%. For women, the optimal cutoff was >2.07 Watt.Kg-1, with a sensitivity of 77.6% and a specificity of 60.4%.

A receiver operating characteristic (ROC) analysis was conducted to evaluate the ability of power to identify frailty / non-frailty classification.”

Comments from Reviewer:

5. In addition, while the coding of the dependent variable (non-frail = 1, frail = 0) is appropriate, a short clarification on how to interpret the resulting odds ratios (i.e., in terms of likelihood of being non-frail rather than risk of frailty) would improve clarity.

Adjustment

In the discussion section, we have amended the section to clarify the results of the Odds Ratio and the direction referred to by the reviewer.

“Our study reported an odds ratio of 1.83 for muscle power predicting frailty, which, when inverted (1/1.83 =55%), means that for each unit increase in power (1 Watt·kg⁻¹), the chance of being frail is reduced by 55%. Although it should be noted that changes of 0.1 -0.2 are more likely to occur.”

Comments from Reviewer:

6. Finally, as estimated muscle power is derived directly from the 5xSTS test, it would be useful to more explicitly acknowledge that the comparison between muscle power and 5xSTS time reflects closely related measures rather than fully independent constructs.

Adjustment

Our Limitations section has been amended to include the following:

“The power presented here is still an estimate, based on individuals' physical performance, using indirect equations rather than a direct measure of the muscles involved in the sit-to-stand action. These are closely related (supplemental data, r = -.82, increased chair stand times are highly but negatively correlated with estimated power) and not fully independent constructs.”

We are grateful to the reviewers and to you for your time and comments in bringing the paper to this stage and look forward to seeing it published.

Kind regards

Cillin Condon

Attachment Submitted filename: Response to Reviewers PLOS v2.docx

# Editor report: Decision Letter 2

**DOI:** 10.1371/journal.pone.0352160.r005

**Authors:** Francesco Curcio, Francesco Curcio, Francesco Curcio

7 Jun 2026

The Role of Estimated Muscle Power from a Sit-to-Stand test in determining Frailty in Community-Dwelling Older Adults.

PONE-D-25-61976R2

Dear Dr. Cillin Condon ,

We’re pleased to inform you that your manuscript has been judged scientifically suitable for publication and will be formally accepted for publication once it meets all outstanding technical requirements.

Within one week, you’ll receive an e-mail detailing the required amendments. When these have been addressed, you’ll receive a formal acceptance letter and your manuscript will be scheduled for publication.

An invoice will be generated when your article is formally accepted. Please note, if your institution has a publishing partnership with PLOS and your article meets the relevant criteria, all or part of your publication costs will be covered. Please make sure your user information is up-to-date by logging into Editorial Manager at Editorial Manager® and clicking the ‘Update My Information' link at the top of the page. For questions related to billing, please contact billing support.

If your institution or institutions have a press office, please notify them about your upcoming paper to help maximize its impact. If they’ll be preparing press materials, please inform our press team as soon as possible -- no later than 48 hours after receiving the formal acceptance. Your manuscript will remain under strict press embargo until 2 pm Eastern Time on the date of publication. For more information, please contact onepress@plos.org.

Kind regards,

Francesco Curcio, M.D., Ph.D.

Academic Editor

PLOS One

Additional Editor Comments (optional):

The authors have satisfactorily addressed the concerns raised during the previous review round. I believe the manuscript is suitable for publication.

Reviewers' comments:

# Editor report: Acceptance letter

**DOI:** 10.1371/journal.pone.0352160.r006

**Authors:** Francesco Curcio, Francesco Curcio, Francesco Curcio

PONE-D-25-61976R2

PLOS One

Dear Dr. Condon,

I'm pleased to inform you that your manuscript has been deemed suitable for publication in PLOS One. Congratulations! Your manuscript is now being handed over to our production team.

At this stage, our production department will prepare your paper for publication. This includes ensuring the following:

* All references, tables, and figures are properly cited

* All relevant supporting information is included in the manuscript submission,

* There are no issues that prevent the paper from being properly typeset

You will receive further instructions from the production team, including instructions on how to review your proof when it is ready. Please keep in mind that we are working through a large volume of accepted articles, so please give us a few days to review your paper and let you know the next and final steps.

Lastly, if your institution or institutions have a press office, please let them know about your upcoming paper now to help maximize its impact. If they'll be preparing press materials, please inform our press team within the next 48 hours. Your manuscript will remain under strict press embargo until 2 pm Eastern Time on the date of publication. For more information, please contact onepress@plos.org.

You will receive an invoice from PLOS for your publication fee after your manuscript has reached the completed accept phase. If you receive an email requesting payment before acceptance or for any other service, this may be a phishing scheme. Learn how to identify phishing emails and protect your accounts at https://explore.plos.org/phishing.

If we can help with anything else, please email us at customercare@plos.org.

Thank you for submitting your work to PLOS ONE and supporting open access.

Kind regards,

PLOS ONE Editorial Office Staff

on behalf of

Dr. Francesco Curcio

Academic Editor

PLOS One
